# A Systematized Review of the Relationship Between Obesity and Vitamin C Requirements

**DOI:** 10.1016/j.cdnut.2024.102152

**Published:** 2024-03-29

**Authors:** Julia K Bird, Edith JM Feskens, Alida Melse-Boonstra

**Affiliations:** Division of Human Nutrition and Health, Wageningen University & Research, Wageningen, the Netherlands

**Keywords:** vitamin C, obesity, vitamin requirements, vitamin status, vitamin intake

## Abstract

Obesity rates have increased globally in recent decades. Body weight is used as a modifiable factor in determining vitamin requirements. Accordingly, vitamin C requirements are volumetrically scaled from data for healthy weight males to other age- and sex-based categories. Likewise, it is possible that increases in body weight due to obesity may affect vitamin C needs. A systematized literature review was performed to summarize evidence on whether obesity affects vitamin C intake or status. The literature was also scanned for potential mechanisms for the relationship. Many observational studies showed that vitamin C status is lower in overweight and obese children and adults; this may be explained by lower vitamin C intakes. Nevertheless, a reanalysis of carefully conducted intervention studies has demonstrated a lower vitamin C status in participants who were overweight or obese when given the same dose of vitamin C as subjects of normal weight. Several mechanisms have been proposed to potentially explain why vitamin C status is lower in people with obesity: changes in vitamin C partitioning between lean and adipose tissue, volumetric dilution, metabolic alterations due to obesity, and gut microbial dysbiosis. Depletion-repletion or pharmacokinetic studies that include individuals of diverse body weights and ages would be helpful to further investigate whether obesity increases requirements for vitamin C. The current evidence base supports a lower vitamin C status in people who are overweight or obese; however, the association may be attenuated by lower vitamin C intakes.

## Introduction

Vitamin C is an essential dietary component required for the normal functioning of many metabolic processes in the body [1]. It is required for the biosynthesis of collagen, carnitine, and various neurotransmitters and is an important water-soluble antioxidant capable of scavenging free radicals. As a reducing agent, vitamin C improves the absorption, transport, and storage of iron. It is also required for normal functioning of the immune system. More recent evidence suggests a role for vitamin C in gene expression and epigenetics: ascorbic acid can regulate hypoxia-inducible factor-hydroxylases [2] and shows epigenetic actions through the regulation of Ten Eleven Translocase DNA demethylases and histone demethylases [3]. Frank deficiency results in scurvy, which is characterized by connective tissue dysfunction, negative mood changes, immune deficits, and muscle weakness and is ultimately fatal. Subclinical deficiency causes fatigue and lassitude [1], affecting quality of life and work performance.

Vitamin C intake requirements for many countries are based on small pharmacokinetic studies performed in healthy young participants with a BMI (in kg/m^2^) in the normal range [4,5]. However, there are indications that people with a higher body weight have lower plasma vitamin C concentrations. Several putative mechanisms may be involved, which we list here and elaborate on further in the article:

1. Vitamin C intakes may be lower in obese individuals due to differences in the pattern of food consumption, which results in lower vitamin C status.
2. The increase in total body size in obesity results in volumetric dilution of vitamin C, which may be partially offset by nutrient partitioning in lean or adipose tissue.
3. Basal metabolic rate increases in obese individuals due to increases in body mass, which affects vitamin C demand.
4. Metabolic alterations occur due to obesity and affect vitamin C status, metabolism, absorption, and/or excretion.
5. Gut microbial dysbiosis due to obesity affects vitamin C absorption or metabolism.

Given that obesity rates and thus body weight, body fat, and incidence of metabolic syndrome have increased in many countries over the past decades [6], the importance of determining the effect of body weight on vitamin C requirements is imperative to avoid subclinical or frank vitamin C deficiency. The aim of this review article is to gather and summarize information on the relationship between obesity and vitamin C requirements.

### Basis of dietary intake recommendations in different countries and regions

Dietary intake requirements have been set to prevent the negative effects of frank or marginal deficiency on health. Different regions and countries have used different criteria to set intake requirements. However, these requirements are extrapolated from studies conducted in young, healthy males to other age and gender groups using bodyweight-based isometric scaling. Body weight is a significant predictor of vitamin C status, and the global increase in obesity rates, therefore, calls into question whether the current requirements are still adequate to meet biological needs if this assumption is, indeed, correct [7].

The World Health Organization sets the reference nutrient intakes (RNI) for vitamin C based on the intake at which body content is halfway between tissue saturation and the point at which clinical signs of scurvy appear [8]. Clinical signs of scurvy appear at a body vitamin C content of 300–400 mg, and tissue saturation was assumed to be 1500 mg for adult males; thus, the halfway point was considered to be 900 mg whole-body vitamin C. There was a lack of data to allow robust interpolation to adult females or children. Therefore, the RNI for females was kept the same as for males, and a graduated increase from recommendations for infants based on human breast milk vitamin C content to that of adults was made for children aged 1–18 y.

The European Food Safety Authority considered fasting plasma vitamin C concentrations >50 μmol/L to be an appropriate target at which the different functions in the body that rely on vitamin C can be fulfilled [4]. The intake required to maintain this plasma level was based on a study of 7 apparently healthy young males of unknown body weight [9]. Although a similar study was performed in 15 healthy nonsmoking females with a mean body weight of 59.1 ± 8.7 kg [10], the data for males were considered the most complete. Hence, the Average Requirements for most other age groups, with the exception of infants for which the basis is the vitamin C content of breastmilk, was extrapolated based on body weight, using 68.1 kg as the reference adult males’ body weight.

The Institute of Medicine considered maximal neutrophil vitamin C content and minimal urinary loss to be the most appropriate biomarkers. Intakes at which these criteria were met that were used for deriving the dietary reference intakes were based on the same study in 7 healthy young males mentioned above [5,9]. Dietary reference intakes are extrapolated to females and children based on bodyweight.

The Australian National Health and Medical Research Council and the New Zealand Ministry of Health based the Nutrient Reference Values (NRV) for vitamin C on the same principles as the World Health Organization, that is, the halfway point between tissue saturation and the appearance of the clinical signs of scurvy [11]. The same NRV was set for males and females, although a difference in bodyweight that may affect requirements was mentioned, and intakes in children aged 1–18 y were interpolated from those for adults.

## Methods

The aim of the review was to gather evidence on the current body of knowledge related to vitamin C intake or status and obesity, including summarizing evidence contained in surveys, clinical trials, mechanistic studies, and reviews where appropriate; thus, the systematized review type was selected [12]. The National Library of Medicine’s PubMed database was searched for the terms “vitamin C” and obesity. The title and abstract were scanned to determine whether the article contained information related to the subject matter. Articles containing data describing vitamin C intake or status according to BMI or obesity status were tabulated, and the results were summarized in the text. Mechanistic evidence was marked according to the mechanism it described and the results used in the discussion of the various mechanisms. Where appropriate, additional searches were conducted, or reference lists were perused for other relevant articles.

## Vitamin C Intakes in Participants with Overweight or Obesity

### Vitamin C intakes and obesity in large and nationally representative surveys

Various studies have looked in detail at the intake of vitamin C in participants who were overweight or obese, both in representative surveys and smaller target groups (Table 1) [13–36]. A large cohort study conducted in 19,068 older adults in the United Kingdom showed an inverse trend between vitamin C intake and waist-hip ratio [20]. In a survey of micronutrient intakes of 6602 adults aged 18–59 y in China according to BMI category, males with obesity had lower vitamin C intakes than the other weight categories, whereas vitamin C intakes increased with increasing BMI in females [18]. The same trend was seen in a longitudinal study of 13,421 Spanish adults over 11-y follow-up, in which participants with the lowest vitamin C intake tertile had the highest BMI [17]. In contrast, data from the Swedish National March Cohort of 43,865 adult males and females showed that there was no difference in the proportion of adults in different tertiles of vitamin C intake between BMI categories [16].

**TABLE 1:** Vitamin C intake and obesity

Reference	Country	Study design	Study population	Dietary assessment method	Mean vitamin C intake (mg/d)	Relationship between vitamin C intakes and obesity
Cohort studies						
Kong et al. [13] (2023)	Korea	Prospective cohort	11,379 adults aged 40+ y	106-item FFQ	Q1 vitamin C intake median: 18.6 Q2 vitamin C intake median: 30.8 Q3 vitamin C intake median: 44.7 Q4 vitamin C intake median: 72.0	Lower risk of developing metabolic syndrome over 5 y in age-adjusted and multivariable models for males.
Jalali et al. [14] (2022)	Iran	Prospective cohort	6248 adults aged 40–70 in Kharameh	130-item FFQ	Normal weight %RDA: 112 ± 51% Overweight %RDA: 124 ± 52% Obese %RDA: 131 ± 56%	Inverse association between risk of obesity and vitamin C intake
Park [15] (2023)	Korea	Hospital-based cohort	58,701 adults aged >40 y from a large city hospital	106-item semiquantitative FFQ	Male, low insulin resistance 107 ± 0.46 Male, high insulin resistance 106 ± 1.22 Female, low insulin resistance 105 ± 0.32 Female, high insulin resistance 99.1 ± 1.22	Vitamin C intakes above 100 mg are protective against insulin resistance.
Hantikainen et al. [16] (2021)	Sweden	Nationally representative cohort	43,865 males and females (18–94 y) from 1997 to 2016	Semiquantitative FFQ	Female Q1 < 80.5 Female Q3 116–618 Male Q1 <64.9 Male Q3 95–321	No difference in the distribution of vitamin C intakes between BMI categories.
Martin-Calvo et al. [17] (2017)	Spain	Longitudinal cohort	13,421 university graduates, mainly from the University of Navarra	Semiquantitative FFQ	T1 148 ± 44.2 T2 257 ± 33.0 T3 445 ± 114	The highest tertile of vitamin C intake had the lowest mean BMI.
Huang et al. [18] (2020)	China	Longitudinal cohort survey	6602 adults aged 18–59 from the China Health and Nutrition Survey	3 × 24-h recall on 3 consecutive days	Males underweight 81.8 ± 9.8 Males healthy weight 81.1 ± 2.5 Males overweight 88.2 ± 3.0 Males obese 73.2 ± 7.2 Females underweight 79.8 ± 14 Females healthy weight 87.5 ± 4.1 Females overweight 91.1 ± 5.9 Females obese 93.5 ±12	Intakes lower in obese males, but higher in obese females.
Bingham et al. [19] (2007)	United Kingdom	Longitudinal cohort	Norfolk EPIC, 404 obese and 471 normal weight adults aged 52–85 y	FFQ	Obese 141 (134−148) Normal weight 127 (121−133)	Vitamin C intake higher in participants with obesity
Canoy et al. [20] (2005)	United Kingdom	Longitudinal cohort	19,068 older adults (45–79 y) located in Norfolk	7-d food diary	Males WHR Q1 86.0 ± 51.2 Males WHR Q2 84.9 ± 45.7 Males WHR Q3 82.2 ± 50.7 Males WHR Q4 79.0 ± 46.6 Females WHR Q1 91.0 ± 49.6 Females WHR Q2 89.1 ± 47.8 Females WHR Q3 85.0 ± 44.9 Females WHR Q4 82.1 ± 45.3	Inverse trend between vitamin C intake and waist-hip ratio.
Cross-sectional studies						
Lin et al. [21] (2023)	Taiwan	National cross-sectional survey	Participants aged >19 y	2 × 24-h recalls one week apart	Males 116.47 (IQR 144.43) mg Females 111.46 (IQR 157.54) mg	Obese females were less likely to meet vitamin C intake requirements.
Betancourt-Nunez et al. [22] (2022)	Mexico	Cross-sectional survey	763 adults aged 18+ working at the University of Guadalajara	Semiquantitative FFQ	AO and no EE 347 ± 225 AO and low EE 296 ± 205. AO and high EE 269 ± 174 No AO and no EE 304 ± 161 No AO and low EE 280 ± 137 No AO and high EE 309 ± 164	Obese participants had lower vitamin C intakes, except for those with emotional eating.
Piyathilake et al. [23] (2022)	USA	Cross-sectional survey	509 females of childbearing age (18–49 y) in Birmingham, Alabama	Block food frequency questionnaire 98.2	Not reported	Females who consumed more than the recommended number of calories were more likely to have an adequate vitamin C intake.
Correa-Rodriguez et al. [24] (2020)	Spain	Cross-sectional	562 young adults	72-h dietary recall	Overweight/obese 70.7 ± 60.6 Normal weight 88.6 ± 72.6	Vitamin C intake was a negative predictor of obesity after adjustment for energy intake, gender, and age.
Eslami et al. [25] (2020)	Iran	Cross-sectional	356 children (7–10 y) in Tehran	FFQ	Total population 174.35 ± 6.76	Children in the highest quartile of the Dietary Phytochemical Index (>214 mg vitamin C per day) had the lowest risk of obesity in crude and adjusted models.
Wlodarczyk et al. [26] (2018)	Poland	Cross-sectional	114 nonsmoking females in Warsaw	2 × 3-d food records	Obese 98 ± 57 Control 120 ± 52	Lower vitamin C intake in subjects with obesity (*P* > 0.05).
Nogueira-de-Almeida et al. [27] (2015)	Brazil	Cross-sectional	126 children (6–18 y) in São Paulo	2-d food diary	Overweight/obese 38.1 (44.6–77.3) Healthy weight 19.4 (30.5–55.9)	Vitamin C intake was higher in overweight/obese children.
Garcia et al. [28] (2012)	Mexico	Cross-sectional	580 adult females (25–55 y)	3 × 24-h dietary recalls	Normal weight 70.0 (27.8, 112.1) Overweight 94.5 (66.4, 122.6) Obese 53.9 (18.2, 89.5)	Vitamin C intake is lowest in obese females.
Mah et al. [29] (2011)	USA	Cross-sectional	16 university students in Connecticut, USA	3-d dietary records	Obese 74.2 ± 13.4 Healthy weight 150.7 ± 34.1	Obese males’ vitamin C intakes were 51% lower than for obese males.
Menzie et al. [30] (2008)	USA	Cross-sectional	207 obese and 177 nonobese adults in Bethesda, Maryland	7-d food records	Obese 75 ± 6 Nonobese 90 ± 7	Vitamin C intake significantly lower in obese compared with nonobese participants.
Kant [31] 2002	USA	Cross-sectional	2765 adolescents aged 12–16 y	1 × 24-h recall	Not reported	Overweight boys more likely to meet vitamin C intake requirements, weight did not affect girls’ vitamin C adequacy.
Gera and Khetarpaul [32] (2000)	India	Cross-sectional	100 obese and 100 healthy weight males aged 35–55 y in Hisar city of Haryana state	3 × 24-h recall on 3 consecutive days (2 working days and a holiday	Obese 137 ± 33.2 Nonobese 101 ± 25.8	Obese males consumed more vitamin C than nonobese males (*P* < 0.05).
Clinical trial baseline data						
McKay et al. [33] (2020)	Australia	RCT baseline data	126 overweight and obese adults	3-d food diary	103.3 ± 6.9	Inverse nonsignificant correlation between vitamin C intakes and BMI.
Song et al. [34] (2019)	USA	RCT baseline data	159 African American adults (18+ y) with uncontrolled hypertension in Baltimore, Maryland	Block Fruit-Vegetable-Fiber Screener	All 100.3 (5.95–222.0) Female 92.1 (5.95–222.0) Male 123.1 (30.4–192.7)	Obesity inversely associated with vitamin C intake in males (BMI >30 kg/m^2^ = decrease in vitamin C intake by 24.5 mg/d).
Netto et al. [35] (2012)	Brazil	RCT baseline data	26 bariatric surgery patients and 26 healthy controls	Semiquantitative FFQ	Bariatric surgery 283 ± 11.8 Normal weight control 242 ± 12.2	At baseline, morbidly obese bariatric surgery patients higher vitamin C intakes than controls.
Case-control studies						
Vahid et al. [36] (2023)	Iran	Case-control	1605 adult patients of Arak Medical Centers, and their caregivers	124-item FFQ	Overweight/obese: 129% ± 58% of RDA Normal weight: 138% ± 64% of RDA	Lower vitamin C intake in overweight/obese group (*P* < 0.01).

Abbreviations: AO, abdominal obesity; EE, emotional eating; FFQ, Food Frequency Questionnaire; WHR, waist-hip ratio.

### Vitamin C intakes and obesity in studies conducted in selected populations

Smaller studies conducted in different population subgroups also investigated vitamin C intakes according to obesity status. The majority show an inverse association between vitamin C intake and obesity. A small study conducted on healthy, young university students in Connecticut, USA, who were lean or obese found that vitamin C intakes were 50% lower in the obese group [29]. A cross-sectional dietary study in a group of 763 Mexican adults with and without abdominal obesity looked at the relationship between vitamin C intake and emotional eating [22]. The results show that vitamin C intakes were higher in participants without abdominal obesity. Participants with obesity who exhibited emotional eating had significantly lower intakes of vitamin C than obese nonemotional eaters, whereas emotional eating in nonobese subjects did not affect vitamin C intakes. This was probably related to a greater proportion of emotional eaters with obesity following the “Snacks and fast food” dietary pattern and a lower proportion following the “Healthy” dietary pattern, which were, respectively, poor and rich in vitamin C-containing foods. A further cross-sectional study conducted on 562 young adults in Spain found that vitamin C intake was an inverse predictor of obesity after controlling for age, sex, and energy intake [24]. In a study conducted on a group of 126 overweight and obese Australian adults, there was a weak inverse correlation between BMI and vitamin C intake (*P* = 0.2) [33]. In a population study from Kharameh province in Iran, data from 6248 participants showed an inverse association between vitamin C adequacy and risk of obesity [14]. Moreover, in Iran, the vitamin C intake of 812 patients with a BMI >25 attending a medical center was compared with 793 normal-weight subjects selected from their caregivers. The overweight/obese group had a significantly lower vitamin C intake than the controls [36].

A convenience sample of 207 obese and 177 nonobese adults in Maryland, USA, found that subjects who were obese had a significantly lower intake of vitamin C, and this was put forward as a potential factor in the hypoferremia of obesity [30]. Baseline data of a randomized, controlled trial of 159 African American adults with uncontrolled hypertension found that obesity was inversely associated with vitamin C intake in the multivariable linear regression, and the relationship was significant for males (*P* = 0.03) but not for females (*P* = 0.30). A case-control study conducted in Warsaw, Poland, with a study group consisting of 114 females with and without obesity, found that the obese group had a lower mean vitamin C intake than the nonobese group [26]. A study conducted on 200 males in India found that males with obesity consumed more vitamin C (137 mg/d) than nonobese males (101 mg/d). This was likely due to the higher consumption of fruit and vegetables by males with a higher BMI [32].

### Gender differences in vitamin C intake according to BMI

Gender differences were found between adults in 2 studies. In an analysis of 16,414 adults in the Korean National Health and Nutrition Examination Survey, females in the highest quintile for vitamin C intake had the lowest risk of abdominal adiposity; however, this relationship was not seen in males [37]. Using data taken from 3075 adult participants of the Nutrition and Health Survey in Taiwan, poorer adherence to vitamin C intake requirements was found in females with obesity but not males with obesity (obesity was defined as BMI >27) [21].

### Life stage differences in vitamin C intakes according to obesity status

Studies in children found that higher vitamin C intakes were associated with obesity. In an analysis of NHANES III data of 12–16-y-old boys and girls, boys who were overweight were more likely to meet vitamin C intake requirements than those in the normal weight range or underweight; however, there was no difference in vitamin C intake according to weight status in girls [31]. Additionally, in children and adolescents in Brazil, children who were overweight or obese had a vitamin C intake that was double that of healthy weight children [27]. Potentially, the availability and consumption of fruit juices that contain vitamin C contributed to the development of obesity in children, although it also improved vitamin C intakes. On the other hand, a cross-sectional study conducted in Tehran, Iran, of 356 children showed that a vitamin C-rich diet high in phytochemicals was associated with a lower risk of obesity in both the crude and adjusted models [25].

### Summary of vitamin C intakes in overweight and obese populations

These studies show an apparent inverse association between obesity and vitamin C intake in adults, whereas the association was positive in children. Children’s fruit juice consumption may increase both vitamin C intakes and increase risk of obesity. A major potential confounding factor in these dietary intake studies was increased underreporting of food intakes in participants with overweight and obesity [38]. The lower vitamin C intakes found in participants with overweight and obesity may be due to underreporting of overall food intakes rather than an actual lower food intake. Furthermore, dietary assessment methods are prone to measurement errors that can distort the results [39]. The studies using an FFQ or diet records are more appropriate for ranking subjects [40]; thus, these results give a good estimate of the relationship between intake and obesity. On the other hand, the 24-h dietary recall studies estimate the population intake more accurately, but the error introduced by daily fluctuations in intake means that the association with obesity may be attenuated [41]. Systemic errors such as social desirability may mean that vitamin C-rich foods, which are generally perceived as healthy, are over-reported.

## Indicators of Vitamin C Status and Obesity

The relationship between vitamin C status indicators and measures of obesity is summarized in Table 2 [19,28,[42], [43], [44], [45], [46], [47], [48], [49], [50], [51], [52], [53], [54], [55], [56], [57], [58], [59]]. One reanalysis of a clinical trial has investigated this relationship. Body weight was a significant predictor of plasma ascorbate concentration within clinical study participants [60]. In this reanalysis, data from 2 clinical studies were pooled. The results show a decrease in plasma ascorbate of 0.263 μmol/L for each increase in body weight (kg). BMI and body weight were collinear; therefore, BMI was not included in the model [60].

**TABLE 2:** Vitamin C status and obesity

Reference	Country	Study population	Study design	Status marker (method)	Reported status	Vitamin C status and obesity
Cohort studies						
Pearson et al. [42] (2017)	New Zealand	404 adults aged 49–51 y living in Canterbury, 47 had low vitamin C <23 μmol/L	Prospective cohort	Plasma vitamin C (HPLC)	44.2 (42.4–46.0) μmol/L	BMI, body weight, and waist circumference significantly higher in low vitamin C adults (adjusted *P* < 0.004).
Cross-sectional studies						
Powers et al. [43] (2023)	USA	13,824	Cross-sectional	Serum vitamin C (chromatogenic)	Healthy 56.1 (51.7–60.5) μmol/L Overweight 55.9 (53.4–58.3) μmol/L Obese 45.4 (42.3–48.5) μmol/L	Obese participants had a lower plasma vitamin C concentration than overweight and healthy weight participants (*P* < 0.0001). Deficiency prevalence higher in obese (7.7%) compared with overweight (4.4%, *P* < 0.05).
Wilson et al. [44] (2017)	New Zealand	89 adults (healthy, with prediabetes, with diabetes) aged 18+	Cross-sectional	Plasma vitamin C (HPLC)	Normal 57 ± 14 μmol/L Prediabetes 48 ± 16 μmol/L T2DM 41 ± 18 μmol/L Total 49 ± 17 μmol/L	Plasma vitamin C concentrations were lower in prediabetes (48 μmol/L), and diabetes (41 μmol/L) patients compared with healthy controls (57 μmol/L); (pre)diabetes patients had a greater body weight, BMI, fat mass, and waist circumference.
Wulaningsih et al. [45] (2017)	USA	7743 adults aged 20+ y (discovery set) and 7988 adults aged 20+ y (replication set)	Cross-sectional	Serum vitamin C (HPLC)	Low-marginal VCS 11.4 μmol/L Normal VCS 41.5 μmol/L Saturated VCS 75 μmol/L (reported in [46])	Inverse correlation between abdominal obesity and serum vitamin C, significant in females (OR 0.8 (95% CI: 0.72–0.89) in discovery set and 0.74 (95% CI: 0.65–0.84) in replication set).
García et al. [47] (2013)	Mexico	197 school-aged children in the state of Querétaro	Cross-sectional	Serum vitamin C (HPLC)	136 (49) μmol/L	Vitamin C concentrations inversely correlated with BMI, body fat %, waist circumference.
García et al. [28] (2012)	Mexico	580 females aged 25–55 y in the state of Querétaro	Cross-sectional	Plasma vitamin C (HPLC)	Normal weight 30.7 ± 13 μmol/L Overweight 30.7 ± 13 μmol/L Obese 29.0 ± 13 μmol/L	No difference in mean plasma vitamin C concentrations between BMI categories; however, the prevalence of low (< 22 μmol/L) and very low (< 11 μmol/L) vitamin C concentrations was higher in the obese and overweight groups, and there was an inverse correlation between vitamin C concentration and BMI.
Pincemail et al. [48] (2011)	Belgium	897 healthy adults aged 40–60 y in Liege	Cross-sectional	Plasma vitamin C (spectrophotometric)	BMI<25 kg/m^2^ 58.5 ± 22 μmol/L BMI>25 kg/m^2^ 54.0 ± 22 μmol/L	Participants with a BMI < 25 kg/m^2^ had a higher plasma vitamin C concentration compared with participants with a BMI > 25 kg/m^2^ (*P* = 0.0026).
Riess et al. [49] (2009)	USA	266 abdominal surgery patients, including 136 laparascopic bariatric surgery patients	Cross-sectional	Plasma vitamin C (HPLC)	BMI 19−25 52.3 ± 22 μmol/L BMI 25–30 46.0 ± 24 μmol/L BMI 30–35 43.8 ± 26 μmol/L BMI 35–40 34.7 ± 19 μmol/L BMI >40 39.8 ± 20 μmol/L	Overweight and obese BMI categories were more likely to have patients with vitamin C deficiency (<17 μmol/l).
Bingham et al. [19] (2007)	United Kingdom	404 obese and 471 normal-weight participants in the EPIC-Norfolk study	Cross-sectional	Plasma vitamin C (spectrophotometric)	Obese 52.4 (50.2–54.7) μmol/L Normal weight 58.7 (56.8–60.6) μmol/L	Subjects in higher quintiles of plasma vitamin C had a decreased risk of obesity (OR Q5 vs. Q1 0.32, trend OR 0.75, *P* ≤ 0.001).
Johnston et al. [50] (2007)	USA	118 sedentary, nonsmoking adults, 54% obese and 24% overweight, recruited from Arizona State University campus community	Cross-sectional	Plasma vitamin C (spectrophotometric)	BMI 18–25 54.4 ± 2.7 μmol/L BMI 25–30 46.4 ± 2.4 μmol/L BMI 30–35 38.3 ± 2.1 μmol/L BMI 35+ 37.2 ± 2.0 μmol/L	Plasma vitamin C was inversely related to BMI, percentage of body fat, and waist circumference in both females and males (r ¼ 20.383 to 20.497, *P* = 0.025).
Galan et al. [51] (2005)	France	1821 females aged 35–60 y and 1307 males aged 45–60 y	Cross-sectional	Serum vitamin C (automated continuous flow)	Males 50.0 ± 22 μmol/L Females 60.2 ± 31 μmol/L	BMI was a negative predictor of serum vitamin C concentration.
Tungtrongchitr et al. [52] (2003)	Thailand	149 overweight/obese and 113 normal-weight volunteers recruited from a Bangkok hospital	Cross-sectional	Serum vitamin C (spectrophotometric)	Overweight 199 (170–267) μmol/L Normal weight 369 (0–1306) μmol/L	Serum vitamin C concentration lower in overweight/obese participants (3.5 mg/L) compared with normal weight participants (6.5 mg/L).
Harnroongroj et al. [53] (2002)	Thailand	270 overweight and obese Thai subjects and 175 normal-weight subjects	Cross-sectional	Serum vitamin C (spectrophotometric)	Overweight/obese 28.4 (26–32) μmol/L Control 34.1 (30.7–37.5) μmol/L	Prevalence of vitamin C deficiency was higher in the overweight/obese group (51.5%) compared with the normal-weight group (41.7%).
Case-control studies						
Zheng et al. [54] (2020)	Europe	22,833 participants randomly selected from the cohort of 340,234 EPIC participants with stored blood and buffy coat samples.	Nested case-control	Plasma vitamin C (HPLC)	Noncases 42.6 ± 19.2 μmol/L T2DM 36.3 ± 18.3 μmol/L	Plasma vitamin C 45.6 μmol/L in normal weight, 40.6 μmol/L in overweight, and 38.5 μmol/L in obese participants.
Amin et al. [55] (2020)	Bangladesh	70 obese females and 70 healthy weight controls in Dhaka	Case-control	Serum vitamin C (spectrophotometric)	Obese 29.8 ± 1.4 μmol/L Control 43.8 ± 1.5 μmol/L	Participants who were obese had lower serum vitamin C compared with normal-weight subjects.
Wardzinski et al. [56] (2018)	Germany	Healthy Caucasian volunteers	Case-control	Serum vitamin C (HPLC)	Obese 55 ± 5 μmol/L Normal weight 75±5 μmol/L	Participants who were obese had lower serum vitamin C (55 μmol/L) compared with healthy weight controls (75 μmol/L), inverse correlation seen with pooled data.
Horn et al. [57] (2017)	Brazil	16 morbidly obese bariatric surgery patients and 16 controls of similar age	Case-control	Plasma vitamin C (spectrophotometric)	Obese, presurgical: 85±10 μmol/mL Obese, 180 d postsurgical: 110 ± 5 μmol/mL Control: 120 ± 10 μmol/mL	Participants who were obese had lower plasma vitamin C before surgery (80 μmol/L), compared with 180 d after surgery (110 μmol/L) or to similar-age controls (120 μmol/L).
Retrospective						
Aaseth et al. [58] (2015)	Norway	441 patients at Oslo University Hospital who underwent gastric bypass surgery and had vitamin concentrations measured	Retrospective	Serum vitamin C (micromethod)	Before surgery 46 (42–51) mmol/L 1-y post op. 62 (60–64) mmol/L 2-y post op. 62 (69–65) mmol/L 5-y post op. 60 (51–69) mmol/L	Vitamin C concentrations increased postoperatively (46.0 nmol/L presurgery to 59.7 nmol/L 5 y postsurgery postsurgery).
Clinical trial baseline data						
Gariballa et al. [59] (2014)	United Arab Emirates	100 patients with diabetes attending a hospital in Al Ain	RCT Baseline data	Not reported (HPLC)	Obese 117 ± 17 μmol/L Overweight 155 ± 28 μmol/L Normal weight 132 ± 68 μmol/L	A trend for lower vitamin C status in obese females but not males.

Abbreviations: T2DM, type 2 diabetes mellitus; VCS, vitamin C status.

### Vitamin C status and obesity in large and nationally representative surveys

Several nationally representative surveys contain evidence that obesity is inversely correlated with vitamin C status. In a comparison of vitamin C status between 2003 and 2006 and the 2017–2018 cycles of the representative NHANES cross-sectional survey, mean vitamin C status was lower in obese participants compared to healthy or overweight participants, although the prevalence of deficiency was similar to the healthy weight group [43]. These results were also found in a similar analysis, looking specifically at the effect of body weight and several other factors on vitamin C status from the NHANES 2017–2018 data cycle [61]. Three different analyses from the EPIC-Norfolk cohort also provide evidence for this finding. An EPIC-Norfolk analysis that used data from 19,068 British males and females found that plasma ascorbic acid decreased as waist-hip ratio increased. The relationship remained after adjustment for age, BMI, vitamin supplementation, smoking status, and socio-economic status [20]. In another analysis of a random sample of 22,833 EPIC-Norfolk participants, including 9754 patients with type 2 diabetes, an inverse association was found between BMI and vitamin C status [54]. A related analysis in the EPIC-Norfolk containing data from 404 obese and 471 normal-weight older adults found that the vitamin C intake of the group with obesity was significantly higher; however, the plasma vitamin C concentrations in the normal-weight group was significantly higher [19]. In Canada, a nationally representative survey conducted on 1615 adults found that serum vitamin C concentrations were significantly lower in the obese group than in the normal weight group [62].

Data from the SU.VI.MAX study conducted in a representative sample of older adults in France (1307 males and 1821 females) analyzed serum vitamin C concentrations according to BMI category and other lifestyle variables. Vitamin C intake was a positive predictor of serum vitamin C, whereas BMI was a negative predictor [51]. In a slightly younger population of 897 adults aged 40–60 y living in Liege, Belgium, participants with a BMI <25 had a significantly higher plasma vitamin C concentration than participants with a BMI >25 [48]. A large number of covariates were screened for their association with abdominal obesity using NHANES III data [45]. In this analysis, abdominal obesity was inversely associated with serum vitamin C concentrations in females and males; however, the association was significant only for females.

## Vitamin C Status and Obesity in Studies Conducted in Selected Populations

Several smaller studies also found that vitamin C status was lower in participants who were overweight or obese. In a study conducted only in subjects with a BMI >25 in Australia (N = 127), there was an inverse correlation between obesity and vitamin C status (correlation coefficient −0.133, *P* = 0.068) [33]. Across the Tasman Sea in New Zealand, the CHALICE cohort study investigated vitamin C status in a group of 404 adults aged 49–51 y living in Canterbury. Low vitamin C <23 μmol/L was associated with greater body weight, BMI, and waist circumference. Another study of 89 adults who were healthy or had prediabetes or diabetes in New Zealand found that 3 measures of obesity (BMI, fat mass, and waist-to-hip ratio) were all inversely associated with vitamin C status [44].

A small, carefully performed study of 17 healthy weight and 13 obese German subjects found lower serum vitamin C in subjects who were obese (55 μmol/L compared to 75 μmol/L), and there was an inverse correlation between BMI and serum vitamin C concentrations when the results were pooled [56]. An analysis of 118 sedentary adults in the United States found an inverse association between plasma vitamin C and measures of obesity (BMI, body fat %, and waist circumference) in both males and females [50]. Obese females had lower serum vitamin C concentrations than normal-weight females in a small survey of 70 participants conducted in Bangladesh [55]. A small study that compared 149 overweight and obese Thai adults with 113 normal-weight volunteers found a significantly lower serum vitamin C concentration in the subjects who were overweight and obese [52]. Another small study conducted in Thailand found a higher prevalence of vitamin C deficiency in 270 adults who were overweight and obese compared with 175 normal-weight subjects [53].

Two smaller surveys conducted in rural Mexico investigated measures of obesity and vitamin C status in healthy females and school-aged children. In the study conducted in females aged 25–55, no difference was found in plasma ascorbic acid concentrations between BMI categories. Linear regression found inverse associations between BMI and waist-to-height ratio with vitamin C status; nonetheless, body fat, waist circumference, or abdominal fat were not associated with vitamin C status [28]. Likewise, in Mexican children, there was an inverse association between vitamin C status and waist-to-height ratio, body fat, and abdominal fat [47]. Other measures of obesity in these 2 groups showed the same inverse association with vitamin C status; however, they did not meet statistical significance [28,47].

### Vitamin C status gender differences and obesity

Gender differences were examined in a study of 100 patients with diabetes in a clinical trial conducted in the United Arab Emirates. Males had a lower vitamin C status in general than females. Females in the healthy weight category had a higher vitamin C status than overweight and obese females. The same trend was seen for males; however, it was less pronounced. Perhaps higher rates of smoking in the male participants may have attenuated the relationship, although the authors did not specifically investigate this effect [59]. On the other hand, the serum ascorbate concentration of United States-based teens (1269 boys and 1385 girls, ages 12–18 y) did not vary between different categories of self-perceived body weight in an NHANES III analysis for either boys or girls. Nevertheless, it is possible that the bias introduced by self-reporting of body weight may have attenuated the relationship [31].

### Vitamin C status in bariatric surgery patients

Bariatric surgery patients often present with vitamin deficiencies, although the effect of weight loss after surgery may be difficult to interpret due to recommendations to take dietary supplements postoperatively. A preoperative analysis of 43 bariatric surgery patients in Recklinghausen, Germany, showed a high incidence of vitamin C deficiency: one-third of participants had plasma vitamin C concentrations below the cut-off for deficiency (<28 μmol/L) [63]. This cut-off is considerably higher than the standard cut-off of 11 μmol/L; hence, the proportion deficient is likely to be elevated compared with the normal criterion.

In 266 patients undergoing abdominal surgery, including bariatric surgery in Wisconsin, United States, vitamin C deficiency was more prevalent in overweight and obese BMI categories despite similar rates of both vitamin C supplementation and fruit and vegetable consumption [49]. Another prospective study of 26 bariatric surgery patients and 26 matched controls in Brazil found that despite higher intakes of vitamin C at baseline, vitamin C concentrations in serum were significantly lower in controls than in patients [35]. The postsurgery results are, however, difficult to interpret. There was an increase in serum vitamin C concentrations 1 y after surgery, which was also matched by an increase in the use of vitamin C supplements. Despite continuation of vitamin C supplement use by most patients 2 years postbariatric surgery, serum vitamin C decreased below baseline concentrations (20.5 μmol/L). The authors cite high vomiting prevalence in the postsurgery group as a potential reason for poorer nutritional status. Another small case-control study conducted in 16 female bariatric surgery patients in Brazil and 16 female nonobese controls found that preoperative vitamin C concentrations were lower than those in controls and likewise 180 days postoperatively. However, vitamin C (60 mg) was provided in the period after surgery [57]. An analysis using pooled samples from ≤441 patients at Oslo University Hospital in Norway found that serum vitamin C concentrations were significantly higher at 1 y, 2 y, and 5 y postoperatively, although the use of multivitamin supplements also increased from 9% to 84% and 70% at 1 y and 5 y postsurgery, respectively [58]. On the other hand, a small analysis of vitamin C status in 61 laparoscopic sleeve gastrectomy patients before and after surgery showed no change in vitamin C status after 6 and 12 months of follow-up despite substantial weight loss [64].

### Summary of effects of obesity on vitamin C status

Taken as a whole, these results indicate that adults who are overweight or obese are more likely to have a lower vitamin C status than normal-weight adults. Furthermore, morbidly obese adults who undergo bariatric surgery have a high prevalence of vitamin C deficiency. However, few of these analyses have attempted to adjust for vitamin C intake, either through foods or dietary supplements, at the individual or group level. It is possible that the main cause of low vitamin C status in people who are obese is low vitamin C intake, as found in the previous section. In addition, it is important to consider the method chosen to measure vitamin C status. Although serum and plasma vitamin C are considered to be equivalent, with the same cut-off point used to designate deficiency, measurement with HPLC is considered to be more reliable than spectrophotometric and enzymatic methods [65,66]. Likewise, the sample handling, storage, and processing procedures affect the stability of ascorbic acid in the sample and should be considered when assessing the reliability of results from particular studies [66].

## Partitioning of Vitamin C in Fat-Free Mass and Volumetric Dilution

Higher vitamin C requirements for males than for females, as seen in pharmacokinetic and cross-sectional studies [9,10,67], have been attributed to greater fat-free mass in males. A comprehensive assessment of the ascorbic acid content of different tissue types in healthy humans has not been performed due to difficulties in obtaining samples; however, it has been assessed in calves, for which endogenous ascorbic acid synthesis is not yet active. A recovery and distribution study of ^14^C-labeled ascorbic acid in 1–6 d old Holstein calves found 5% lower vitamin C content in adipose tissue compared with neck muscle tissue and 10% lower compared with the blood 1 wk after an intravenous dose was given [68]. This analysis showed a greater affinity for ascorbic acid in the blood and muscle tissue than the adipose tissue.

In humans, females with replete vitamin C status reached a much higher vitamin C concentration after an identical acute dose of ascorbic acid than males, and the fat-free mass of males was found to be larger [69]. Two small pharmacokinetic studies found that the body’s vitamin C pool was related to the size of the fat-free mass [70] or correlated inversely with body weight [71]. In an analysis of a cross-sectional survey of 270 elderly males and females, fat-free mass was considered an inverse predictor of vitamin C status. There were strong indications of confounding between sex and absolute fat-free mass, although fat-free mass as a relative percentage of total mass, was not tested for confounding [67]. The same group also conducted a longitudinal analysis of>20 y with 399 subjects aged >60 and found that there was an inverse correlation between fat-free mass and vitamin C status in females but not males [72]. In contrast, a 6-mo resistance training program in 57 elderly subjects that modestly increased fat-free mass and reduced fat mass seemed to further increase circulating vitamin C concentrations compared with the control group [73]. The effect of aging on fat-free mass is an additional factor that is not often considered in investigations between fat-free mass and vitamin C status. More focused research into the role of body composition on nutrient partitioning would be helpful in elucidating whether vitamin C concentrations are higher in fat-free mass.

## Basal Metabolic Rate and Vitamin C Requirements

Increased turnover of vitamin C may occur due to oxidative stress from the increase in metabolic rate in subjects who are obese [74]. We were interested in whether there were indications of an increase in vitamin C requirements based on basal metabolic rate rather than metabolic changes due to obesity. Extremely limited research has investigated this relationship. Two human intervention trials looked at changes in metabolism after an intervention that increased vitamin C status and found no effect. One study compared 16 nonobese middle-aged males who were given an energy-restricted diet (80% energy content compared with a weight-maintaining diet) with 8 control subjects. Over the course of the 10-wk intervention, and despite a similar micronutrient content of the diet, the vitamin C status of the energy-restricted group increased from 51.6 μmol to 64.9 μmol, whereas there was no change in the control group (54.3 μmol at baseline to 52.3 at the end of study). Although body weight decreased in the energy-restricted group by 7.4 kg compared with 2.1 kg in the control group, there was no change in the resting metabolic rate measured by indirect calorimetry [75]. Thus, the increase in vitamin C status appears to result from a factor related to a reduction in energy intake and its consequences, excluding metabolic rate changes. In the second study, a seed extract of the African mango plant *Irvingia gabonensis* increased vitamin C status after 12 wk of supplementation; however, there was no change in fat oxidation rate, body weight, energy expenditure, or ad libitum energy intake [76]. The dose of vitamin C in the extract was unknown. These studies did not set out to directly assess whether vitamin C requirements are influenced by metabolic rate, and there is a clear lack of research in this area.

## Metabolic Alterations Due to Obesity

### Chronic inflammation/oxidative stress

Negative health effects of obesity are driven by chronic inflammation [77], which potentially increases the demand for antioxidants [78]. In some countries, smokers are advised to increase their intake of vitamin C to counteract greater losses due to greater metabolic turnover of the vitamin [5]. Both obesity and cigarette smoking increase concentrations of the proinflammatory C-reactive protein (CRP), whereas high dietary intakes of vitamin C appear to decrease it [78]. For example, a direct comparison of CRP concentrations between normal weight, obese, and metabolic syndrome patients showed elevated concentrations in the obese and metabolic syndrome groups [79]. A reanalysis of adults not receiving supplements in 2 large epidemiological studies, NHANES (n = 2828) and EPIC-Norfolk (n = 20,692), showed that the dose-response between vitamin C intake and status was muted in participants with elevated CRP [80]. Similarly, excess adipose tissue was associated with elevated proinflammatory adipokines and lowered anti-inflammatory adipokines [77]. Along these lines, it is possible that the increase in inflammation found in obesity increases turnover of vitamin C, leading to a decrease in vitamin C status and, ultimately, higher requirements.

### Insulin resistance/metabolic syndrome

Associations between vitamin C status and metabolic markers provide some evidence that metabolic alterations due to obesity and vitamin C status may be biochemically linked. Several cross-sectional surveys have investigated the relationship. The dual analysis of NHANES and EPIC-Norfolk data discussed in the section on chronic inflammation found that vitamin C status was lower in participants with diabetes than in those without [80]. When the dose-response relationship between vitamin C intake and status was stratified by diabetes status, the diabetes patients required a higher vitamin C intake to achieve the same status in this study [80]. Inverse correlations were seen between vitamin C status compared with blood pressure, postprandial glucose and insulin, HbA1C, homeostasis model assessment, triglycerides, and free fatty acids in a study of 253 participants who were overweight or obese [64]. Vitamin C concentrations were lower (31.1 vs. 57.8 μmol/L), and prevalence of deficiency was higher (79.6% vs. 8.16%) in 191 patients with the metabolic syndrome, compared with 98 healthy controls in a case-control study conducted in the Medical University of Łódz, Poland [81]. A case-control analysis of 46 adults with diabetes and 42 adults without diabetes in Nigeria found that patients with type 2 diabetes were more likely to be overweight and more likely to have low vitamin C levels despite no difference in vitamin C intakes than the control group [82]. Furthermore, BMI, time with diabetes, and fasting plasma glucose concentrations had an inverse relationship with serum vitamin C concentrations [82].

The results from these studies suggest that there is an association between metabolic syndrome and lower vitamin C status. Nevertheless, studies investigating interactions between the metabolic syndrome and vitamin C requirements through epidemiology are likely to be confounded by the effects of higher BMI or body weight on the burden of metabolic disease, as was seen in several studies [44,81]. Also, there is confounding between metabolic syndrome, obesity, greater body size, and lower vitamin C intakes [83]. Therefore, research looking into whether the metabolic syndrome mediates or exacerbates associations between obesity and vitamin C status must account for these potential confounding factors.

### Increased excretion due to diabetes

As part of vitamin C homeostasis, vitamin C is excreted in the urine when circulating concentrations exceed a certain threshold. Some researchers have identified abnormal excretion of vitamin C in people with diabetes as a potential cause of low vitamin C status [84]. To investigate the renal leak, the vitamin C renal threshold was determined based on data from 2 depletion-repletion studies [9,10], which is the vitamin C concentration above which vitamin C is excreted in the urine. Then, the minimum elimination threshold (MET) was calculated to define a cut-off whereby vitamin C in urine would be considered abnormal, set at 2 standard deviations below the renal threshold. The renal threshold and MET were calculated for each sex. To calculate the prevalence of renal leak, a cross-sectional study was performed with 162 people with diabetes, compared with control subjects without diabetes. The prevalence of renal leak was higher in the diabetes group (33%) than in the control group (9%). There were other differences between the 2 groups: the control group had a higher proportion of females, were younger, and had a lower BMI. Measurements related to kidney function showed that people with diabetes were more likely to have abnormal function. Taking the dataset as a whole, sex, and age were not associated with renal leak, whereas BMI, fasting glucose, hemoglobin A1c, and history of micro/macrovascular complications, which are all related to diabetes severity, were associated with renal leak of vitamin C.

### Gut microbiota dysbiosis

Traber et al. [85] propose a complex relationship between obesity and vitamin C status driven by chronic inflammation and gut microbial dysbiosis. Gut dysbiosis found in obesity compromises gut barrier function, and endotoxins such as bacterial-origin LPS leach into circulation, increasing chronic inflammation. Both increased vitamin C turnover and inhibited absorption result: hypochlorous acid produced by neutrophils under chronic inflammation directly depletes ascorbic acid [86,87], whereas endotoxins in the gut inhibit vitamin C absorption [88,89]. The inverse correlations between vitamin C status and concentrations of inflammatory molecules that we discuss above provide some evidence supporting this theory.

## Conclusions and Recommendations

The current evidence base supports the observation that vitamin C status is lower in people who are obese. A small number of intervention studies show that the dose-response relationship between vitamin C intake and status is blunted in participants who are obese or have a high body weight. Cross-sectional dietary studies mostly support lower vitamin C intakes in people who are overweight or obese, which adds a confounding factor to the body of observational research that found an inverse association between obesity and vitamin C status. An altered metabolic state due to obesity may increase requirements either due to reduced absorption or increased excretion of vitamin C as found in people with diabetes, or the influence of oxidative stress or inflammation. The involvement of the gut microbiota in vitamin C status in obesity is intriguing; however, carefully designed studies in humans are required to ascertain a cause/effect relationship. Pharmacokinetic studies conducted in a more diverse population to include variations by age, body weight, and the presence of the metabolic syndrome would be beneficial to investigate the effect of vitamin C supplementation on vitamin C status, and therefore vitamin C requirements, in overweight and obese adults.

## Author contributions

The authors’ responsibilities were as follows – JKB performed the literature search, wrote the initial draft, and had primary responsibility for final content; AM-B performed project oversight, editing, and discussion of the manuscript content. EJMF performed editing and discussion of the manuscript content. All authors have read and approved the final manuscript.

## Conflicts of interest

The authors report no conflicts of interest.

## Funding

The authors reported no funding received for this study.
